# Harnessing rural community savings groups for contribution collection in the Zambia national health insurance scheme: an analysis of community perspectives using the motivation and ability framework

**DOI:** 10.1186/s12913-026-14721-w

**Published:** 2026-05-20

**Authors:** Adam Silumbwe, Nangana Simataa, Joseph Mumba Zulu, Maio Bulawayo, Mwmba Chewe, Peter Hangoma

**Affiliations:** 1 Department of Health Policy and Management, School of Public Health, University of Zambia, Lusaka, Zambia; 2 Department of Epidemiology and Global Health, Umeå University, Umeå, 901 87 Sweden; 3 Chr. Michelson Institute, P.O. Box 6033, Bergen, N-5892 Norway; 4 Bergen Centre for Ethics and Priority Setting in Health, University of Bergen, P.O. Box 7804, Bergen, N-5020 Norway

**Keywords:** National health insurance, Informal sector, Saving group, Universal health coverage, Zambia

## Abstract

**Background:**

Many countries seek universal health coverage through national health insurance schemes based on payroll contributions from formal sector employees. Zambia, having recently adopted such a scheme, faces challenges in collecting contributions from its large informal sector. Expanding insurance to this sector requires trust and private information, qualities often found in community-based organizations such as savings groups (SGs). This study explores stakeholder perspectives on the feasibility of leveraging SGs for contribution collection from the informal sector in Zambia’s national health insurance scheme.

**Methodology:**

We conducted an exploratory qualitative research study in two districts of Zambia, comprising nine focus group discussions with community members and eight key informant interviews with stakeholders. We applied thematic analysis using the motivation and ability framework, which stipulates several dimensions of assessing feasibility including the triggers, abilities, motivation, and action.

**Results:**

Participants indicated that there were existing “insurance” features in the SGs that could be leveraged to make members appreciate the concept of health insurance. They emphasized the importance of enhanced knowledge about the national health insurance scheme at community level as a key trigger to facilitate engagement of the SG members. The ability of these groups to be used for contribution collection was strengthened by the presence of a collective constitution governing all members. Furthermore, the results revealed a promising opportunity to utilize mobile technology to collect contributions from rural communities. Notably, the motivation within the community was underscored by the recognition of social advantages emanating from the SG and the members’ eagerness to contribute towards emergencies such as healthcare. However, participants identified potential threats to using the SGs for contribution collection, including inconsistent income, mishandling of contributions, and instability within the SGs.

**Conclusion:**

Our study suggests the feasibility of utilizing SGs for contribution collection, yet their effective use may necessitate government oversight, policy development, and capacity building. Interventions such as proper financial management and technology integration can optimize the potential of leveraging SGs for inclusive healthcare coverage.

**Supplementary Information:**

The online version contains supplementary material available at 10.1186/s12913-026-14721-w.

## Background

Global efforts to strengthen health systems are currently centered on advancing universal health coverage (UHC), which aims to ensure that individuals have access to a comprehensive range of high-quality health services without facing financial hardship [1]. This objective is underscored by Sustainable Development Goal 3, target 3.8 [2]. However, low- and middle-income countries (LMICs) encounter challenges in establishing effective health systems financing models to protect citizens from catastrophic health expenditures while ensuring the provision of quality services [3]. Many LMICs rely on out-of-pocket (OOP) payments to finance healthcare [4], which disproportionately affects the poor and contributes to disparities in health outcomes within communities [5]. To address this issue, LMICs are increasingly turning to pre-payment systems, such as national health insurance (NHI), to mobilize financial resources for health systems without exposing individuals to financial risks [4]. NHI schemes are social programs that collect contributions from citizens, regardless of their socioeconomic status, to provide access to healthcare services in times of illness. Several countries, including Ghana, the Philippines, Vietnam, the Republic of Korea, and Kenya, have implemented social or national health insurance schemes as part of their efforts to achieve UHC [6–9].

However, these schemes also experience challenges, particularly when it comes to collecting contributions from the informal sector [10]. The informal sector comprises of entities which operate outside government laws and regulations, characterized by casual labor, self-employment, unregistered businesses, and lack of formal structures [11]. This sector has proved difficult to cover using the voluntary prepayment mechanisms as it is less well-off compared to the formal sector and thus has a lower ability to pay [12]. There are several reasons for low coverage of the informal group. Firstly, the heterogeneity of informal workers makes them difficult to define, identify, and engage in health insurance [13]. Secondly, the labor relationship in the informal sector is based primarily on casual employment, kinship, or personal and social relations, and not on contractual arrangements; thus, informal sector workers often do not have employment security and social security [14]. Lastly, the irregularity of informal sector employment makes it difficult to assess their income for health insurance contributions [8].

Strong penetration in the informal sector requires high levels of trust and private information of people´s economic circumstances. Evidence suggests that community-based organizations (CBOs) have high levels of trust from community members and not using them in implementing health insurance may be a missed opportunity [15]. For example, in India, CBOs that are spread across the country and have built trust in communities are seen as potential conduits for expanding health insurance [16]. Several studies have explored questions of expanding insurance coverage to the informal sector [15, 17, 18]. However, there is a gap in terms of studies that look at how CBOs can be leveraged to expand coverage.

In this study, we focus on Saving groups (SGs), a common form of CBOs in most countries in Africa and Asia [19, 20]. They are formed by community members who agree to save part of their earnings and have their own guiding principles. They have different functions, including providing credit to members with minimum interest, funeral grants, and giving cash to members when they are sick [21]. Evidence has shown that SGs have functioned quite well in improving social welfare services, including improving the capacity to save for emergencies among community members [22]. In terms of healthcare, community members have reported to have used part of these savings for transportation to health facilities, purchase of food and other supplies [21]. However, there is little evidence on how SGs can and should be used in the collection of premiums towards financing the NHI.

### The Zambian context of healthcare financing and insurance coverage

In Zambia, healthcare is mainly financed through general tax, donor grants, and direct OOP obtained from households [23]. Even though the Zambian government abolished user fees for certain health services, some patients are still required to make OOP to access some services in the public sector [24]. According to the 2015 living conditions monitoring survey report, 88.7% of Zambian workers are in informal employment, accounting for more than half of the country’s economic activity. Although there is a slight difference in informality between rural and urban areas, it remains relatively high, standing at more than 85% and 65%, respectively. This translates to 15% of rural and 35% urban residents in formal employment. As a result, the insurance penetration in Zambia is low, at about 2.5% of the gross domestic product, indicating limited coverage of insurance services. General insurance is the most common covering motor vehicles, property and liability for businesses and asset holders [25]. There are also life insurance products that provide funeral and education cover.

The Zambian government has embraced the global movement towards UHC, with a national vision of ‘providing care to all without leaving anyone behind’ [26]. To this effect in 2018, the government initiated the NHI by passing the National Health Insurance Act No. 2 of 2018 [27]. The Act guides that 1% contribution should be deducted every month from those in formal employment. In 2019, a board responsible for operationalizing the NHI was appointed. In the same year, the board approved the benefit package, begun collecting contributions from the formal sector and accrediting health facilities across the country. This scheme was rolled out in two phases with the first part covering the formal sector while the second, extended to the informal sector. However, it remains unclear how contributions collections could be maximized from the informal sector, particularly low-income households at community level.

Across Zambia, SGs such as village saving groups, village banks, or village savings and loan associations are common forms of CBOs in both urban and rural areas, particularly in areas where formal banking services are absent. They offer a viable option for collection of health insurance contributions from the informal sector as people in this sector lack regular salaries or structured payroll systems. Using SGs for contribution collection may allow low-income households to make small and manageable payments that align with their irregular incomes, which potentially, may enhance compliance and access to health insurance.

Whilst the Zambia NHI scheme is unclear on how contributions can be collected from informal local communities; there is indeed an opportunity to leverage on SGs. Therefore, our study, using the motivation and ability framework, explores stakeholder perspectives on the feasibility of leveraging SGs for contribution collection from the informal sector in Zambia.

## Methods

### The motivation and ability (MOTA) framework

**Fig. 1:**
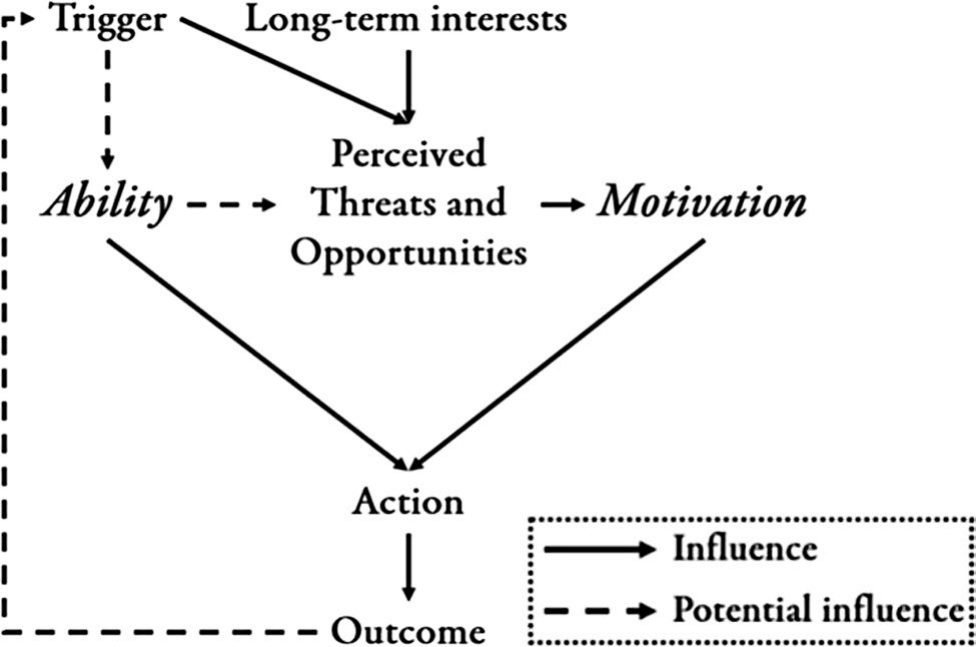
MOTA framework (based on: Phi et al. 2015)

In this study, we adopt the MOTA framework proposed by Phi et al., 2015 (Fig. 1). This framework is particularly useful when seeking to understand factors shaping why community members may adopt (or fail to adopt) the idea of using SGs for contribution collection. It unpacks possible change or action by community members into motivations and abilities [28]. Motivations signify the subjective side of behaviour, the interests, attitudes, and perceptions towards health insurance by the community members. Abilities refer to the resources, means and opportunities community members have to act in their interests or the resources they lack to translate interests into effective actions. Motivation and ability are properties of community members, which can be “triggered” to initiate change. Triggers can be new policies or new supporting tools. It is important to note that the same trigger may have different consequences for the motivations and abilities of different community members, as the trigger might be perceived as a threat, or as an opportunity [28]. The framework also provides a feedback loop, as the outcome of an action can result in a trigger, causing a change in perceptions and abilities.

### Study design

We used an exploratory qualitative study design, building on the absence of previous studies that have examined the use of SGs for contribution collection [29]. This design facilitated an in-depth exploration of the SGs and the social context in which they operate in rural settings through multiple data collection approaches. It also allowed us to develop a more nuanced understanding of the feasibility of using SGs as a mechanism for contribution collection.

### Study setting

The research study was conducted in the rural parts of Southern Province of Zambia. Firstly, in Choma District, three communities were selected: Chishingwa, Macha Central and Nalituba. Secondly in Kalomo District: Chilala, Bbelow and Mutare communities were included. In both districts, the study communities or villages were purposively chosen based on the presence of SGs supported by two local non-governmental organizations (NGOs) – the World Vision International (WVI) and Churches Association of Zambia (CHAZ).

### Study population

The study population consisted of both males and females, 18 years and above, from local communities who were members of the SGs and belonged to the informal sector. In addition, SGs field coordinators who were trained by WVI and CHAZ, government officials from the Ministry of Community Development and Social Services in the two study districts, and staff from the National Health Insurance Management Authority (NHIMA) in Lusaka, participated in this study.

### Sampling and recruitment

Individual participants were recruited using purposive sampling from the registers of SGs managed by WVI and the CHAZ. The local ward development committee leaders, who are a government unit responsible for overseeing savings and loan groups, under the Ministry of Community development and Social Services facilitated recruitment of community members who participated in this study. Similarly, Key informants from the NHIMA, WVI and the CHAZ were purposively recruited based on the role they played in managing the NHI and supervising the SGs, respectively.

### Data collection

Data were collected using key informant interviews (KIIs) and focus group discussions (FGDs) which were audio recorded, and field notes were taken on non-verbal cues to ensure accurate information was captured. The data collection took place between July and November 2022. It commenced with the training of research assistants and piloting of the data collection instruments. The KIIs and FGD guides were developed by the research team based on reviewing existing literature on community informal financial groups (supplementary file). The questions focused on what community members knew about health insurance, the dynamics within the SGs including their advantages and disadvantages, community attitudes and practices towards SGs, and the feasibility of using the SGs for contribution collection. Further, the interview guides for the FGDs were translated in the local language of Tonga. We also ensured that research assistants that we recruited were fluent in the local language.

### Key informant interviews (KIIs)

The various respondents for KIIs included key stakeholders from the two NGOs and the field coordinators. These field coordinators were selected from among the community members and trained by the NGOs to oversee community SGs’ activities. All KIIs with the coordinators were conducted in the communities and those with stakeholders were done at their respective offices. The KIIs were both face-to-face and by phone, due to the COVID-19 situation at the time of data collection. In total, eight (*n*=8) KIIs were conducted (Table 1).

**Table 1:** Participating stakeholders for the KIIs

Participants	Number of interviews
**Stakeholders**	
National health insurance management authority	1
Ministry of social warfare	1
World Vision International	1
Churches Health Association of Zambia	1
Financial sector deepening	1
**Field coordinators**	
Chilala field coordinator	1
Bbelo field coordinator	1
Macha field coordinator	1
**Total number of interviews**	**8**

### Focus group discussions (FGDs)

The FGDs were conducted with community members in Tonga, a local language. Three (*n*=3) FGDS were conducted in Choma District and six (*n*=6) were done in Kalomo District, making a total of nine (*n*=9) groups. Slightly more FGDs were done in Kalomo because it had many SGs registered under both the WVI and CHAZ (Table 2). Each FGD consisted of 8–11 members, resulting in a total of 88 participants.

**Table 2:** Community focus group discussions

District	Area	Number of FGDs	Total Participants
Choma	Macha central	1	9
	Nalituba	1	9
	Chishingwa	1	10
Kalomo	Bbelo village	2	16
	Mutare village	2	22
	Chilala village	2	22
	**Total**	**9**	**88**

### Data analysis

All audio recordings and summaries from the KIIs and FGDs were transcribed verbatim and then transferred to NVivo Pro Software for coding and analysis. The recordings that were captured in the local language were back translated in English by an expert. Thematic analysis, which is a flexible and popular method of qualitative data analysis of grouping sections of data into themes to uncover underlying relationships and patterns was used to analyze data [30]. The data analysis process started with the first and second authors familiarizing themselves with the data by reading of a selected sample of transcripts to generate initial codes and develop some themes. A qualitative data coding framework was then developed after iterative discussions with the rest of the research team and imported to NVivo 12 Pro Software (Table 3). The first and second authors coded all the transcripts and discussed any modifications and updates to the coding with the entire research team. The emergent themes from the data were grouped around the different elements of the study framework as shown below.

**Table 3:** Major themes and sub-themes

Main themes	Sub-themes
1. Triggers that facilitate leveraging community saving groups.	• Enhanced knowledge and understanding of the national health insurance scheme.
2. The ability of community saving groups.	• Availability of a social fund • Collective constitution to govern members
3. Threats to using saving groups.	• Inconsistent income among saving group members • Mishandling of financial contributions within saving groups • Instability of community saving groups
4. Opportunities in using saving groups.	• Leveraging mobile technology to gather contributions from rural communities.
5. Community motivation to use saving groups.	• Belonging to savings group yields various social advantages. • Community members eager to contribute to cover illness.

### Ethics approval and consent to participate

The study was approved by the University of Zambia Biomedical Research Ethics Committee (UNZABREC) and the Zambia National Health Research Authority (NHRA) (REF. No. 774–2019). Approval was also sought from the Ministry of Health Permanent Secretary. During data collection, participants were given information sheets with information on the study accompanied by thorough explanations, to ensure they made informed decisions, before consenting. Written informed consent was obtained from all the study participants. To ensure confidentiality, all participants were given identity numbers which were used during data collection and analysis. Covid-19 guidelines were strictly observed during the study including social distancing, and provision of masks and hand sanitizers. This study was conducted in accordance with the Declaration of Helsinki [31].

## Results

We used the elements of the study framework to organize our findings. Several key themes emerged from the data. The participants underscored the significance of enhancing knowledge about the national health insurance scheme at community level as a key trigger to facilitate engagement of the SG members. The ability of these groups to be used for contribution collection was strengthened by the presence of a social fund and a collective constitution governing members. However, potential threats, including inconsistent income, mishandling of contributions, and instability within SGs, were identified. Furthermore, promising opportunities were revealed, particularly in utilizing mobile technology for contributions from rural communities. Notably, the motivation within the community to participate in SGs was underscored by the perceived social advantages and the members’ willingness to contribute towards healthcare coverage.

## Triggers that can facilitate leveraging community saving groups for contribution collection

Triggers include climatic events, new policies, or supportive tools such as awareness creation efforts that stimulate community members to adopt positions with regard to using SGs for contribution collection. It's crucial to acknowledge that the same trigger may have diverse positive or negative consequences on community members' actions.

### Enhance knowledge and understanding of the national health insurance scheme

The key informants and community members emphasized that enhancing education and awareness about the national health insurance scheme in rural areas would be a crucial factor in facilitating the utilization of SGs for contribution collection. One key informant from Kalomo underscored the need for education and awareness as follows.

*It needs a lot of efforts to educate community members on NHI insurance the same way they are doing on Covid-19 period they are putting in a lot of efforts to educate people*,* and everyone is understanding the protection measures even people living in the village. This is the effort needed by the government to educate people for them to understand health insurance [KII 007*, Kalomo*]*

Moreover, certain community members expressed concerns about the inconsistency of information dissemination. Some received information from the radio, while others obtained it from informal sources in the streets. This led to the spread of distorted and occasionally false information about the national health insurance scheme within the communities. An FGD participant from Choma has this to say.

*I heard of it; I found people chatting and so that is how I got to know about it. They were saying that if a person is in formal employment*,* they were deducting money from their salary. And if a family member like child falls sick it’s very easy for the child to get medical attention because the child is on the scheme of the parent*,* you have no difficulties in getting medical care because the child is already covered on the scheme. I heard people discuss that without knowing the details contained in it [FGD 002*,* Choma]*

## The ability of community saving groups to support the national social health insurance

Abilities include the resources, means, and opportunities available to community members, which determine their capacity to act in their interests. Alternatively, ability reflects the resources they lack, hindering the translation of interests into effective actions.

### Availability of a social fund

The participants mentioned that the SGs had a component called social fund which operated on a similar principle as the health insurance scheme of collecting contributions towards eventualities. The social fund was a form of insurance for members in case of sickness or bereavement. A community member recounted how she got assisted using contributions from the social fund in time of sickness.

*Personally*,* when I got sick and I was admitted*,* they brought money for me to use at the hospital. I bought food stuffs*,* some medical supplies*,* and other items for use. It is not only me but even other members when they are sick. They are given money to use whilst in hospital and it is very helpful [FGD 007*,* Kalomo]*

Some key informants suggested that building on the social fund principle, the contributions could be extended or channeled towards the health insurance scheme. However, for this to be possible, there was a need to not only review how the social fund operates, but also, clarify the mechanisms by which funds could be channeled towards the national health insurance scheme. One key informant from social welfare ministry retorted.

*Social fund is a form of internal group insurance. Those are the funds that the group uses to give to the sick member which is taken from the contributions. So perhaps within the context of the social funds we can extend to NHI. It’s an issue of expanding on that concept of the social fund. Maybe increasing the amount that is being contributed towards the social funds so that money can be remitted to NHI. I think it is a good way of mobilising funds towards the NHI [KII 002*,* National*,* Lusaka]*

### Collective constitution to govern members

According to the key informants, SGs were self-managed groups of like-minded individuals who met monthly to save and borrow. Each group devised its own constitution and structure to govern their operations. The group constitution is a key document that outlines the rules of engagement, organizational structure, authority and responsibilities of individuals, as well as important dates and activities. The constitution further caped the savings – the money loaned to members at a stipulated interest rate and repayment period. A community member had this to say.

*Savings groups are groups of people who come together. This can be a group of 10 to 30 people who mutually agree. The select themselves*,* save and make a pool of funds. Then the give each other loans in which they repay with a minimum interest and share out at the end of the cycle. The members save*,* borrow*,* and have a group constitution to guide how these activities are undertaken [FGD 003*,* Choma]*

In addition, the key informants explained that the SGs had committees which spearhead the running of the groups. The members selected a committee comprising of five representatives. The committee ensured that each member who belonged to the SG formalized their allegiance to the constitution and membership by signing a form. The stakeholders emphasized that adherence to the group constitution was mandatory, and failure to do so would result in deregistration or penalties.

*The operation of the group is that they members are managed by a 5-member committee group*,* selected amongst themselves*,* we have the chairperson of the group*,* the record keeper or the secretary*,* the box keeper and the two money counters. They have a hard copy cash book*,* a ledger book*,* the cash box*,* and the group is encouraged to have its own constitution upon formation. Every member of the group signs on the constitution to become a member [K*II 004, Kalomo*]*

## Threats to using saving groups for contribution collection

Threats may include all factors within the design and function of the SGs that are likely to be potential cause of problems in efforts to use them for contribution collection. These include inconsistent incomes, mishandling of financial contributions and instability.

### Inconsistent income among saving group members

A majority of the rural community members relied on farming for their sustenance, occasionally facing challenges such as droughts. Insufficient harvests often led to significant disruptions in local incomes, resulting in lower savings levels and impacting livelihoods. Further, some community members argued that a significant portion of their earned income was typically allocated towards purchasing farming inputs, which were reported to have seen price increases in recent years. However, the community members still believed that the prepayment mechanism within the SGs was beneficial due to the unpredictable nature of their income. A community member from Choma had this to say.

*Because of the drought we had*,* we experienced hiccups towards saving as our demands were for us to survive*,* to buy food*,* because we could not raise funds to save. But now things are fine/ok we have managed to harvest our crops and intend to commence savings [FGD 002*,* Choma]*

### Mishandling of financial contributions within saving groups

Regarding fund management in the SG, community members expressed the need to prevent pilferage. They indicated that the temptation for people in leadership to misapply their contributions was high, and so the need for preventive mechanisms. Indeed, some participants narrated that some SGs in other places had been disbanded because of mismanagement of funds. This according to the community members was the biggest threat to using the SGs for contribution collection in the national health insurance. A community member from Kalomo had this to say.

*I think the biggest threat will be pilfering or loss of money along the way*,* so you really have to have a proper system that can track the contributions to the lowest unit of the group*,* which is an individual or a family. This can be done through encouraging members to deposit their savings in a mobile money account of the group*,* by providing proof of deposit of contribution. I think that is a very good opportunity especially if it is sold well and understood. People can see the benefits. The other thing we have been trying to do is save without cash but use mobile money [FGD 006*,* Kalomo]*

### Instability of community saving groups

Some key informants shared that the SG structures were not permanent and operated on a cycle-based saving system. They reported that members disbanded after completing a specific saving cycle to facilitate both incoming and outgoing mobility. The concept is that group members can choose to continue operating with the same members based on their performance in the previous cycle, or they may decide to remove certain problematic members. The cycle-based saving system eventually leads to the dissolution of SGs, ultimately resulting in instability. This could potentially hinder their effectiveness for contribution collection in the national health insurance scheme. One key informant expressed.

*The model of these groups is cycle based. The idea behind disbanding is to allow for inward and outward mobility of members. It’s like they are not tied together. Otherwise*,* it would be a bit difficult for groups to continue*,* for example if in a group they had people who defaulted. The members who defaulted need to leave the group because the group says they don’t want them. But as an option if there is a particular service that can be enjoyed in the group but only for the purpose of contributing to the NHI. Then probably they can stay in the group but only for the purpose of contribution*,* not enjoying the other services [KII 001*,* National*,* Lusaka]*

## Opportunities in using savings groups for contribution collection

Opportunities refer to conditions or favorable openings that can enhance the use of SGs for contribution collection. We identified one critical factor relating to mobile technology and its potential use in contribution collection from the informal sector.

### Leveraging mobile technology to gather contributions from rural communities

Some key informants suggested that using the SGs for contribution collection would enhance leveraging of mobile technologies such as mobile money, e-wallets, and other mobile banking services. Providing community members with the latest financial mobile technology options would enable them to conduct quicker financial transactions, reducing the need for physical handling of money and thereby preventing potential mismanagement of contributions by individuals as one key informant indicated.

*Maybe the problem can be solved by using mobile money or e-wallets. This is because cash has all sorts of risks as it brings among other things the temptations to collect payments using cash as somehow it tends to grow legs. So*,* I think to have an effective collection mechanism*,* it would have to be from the individual’s mobile money into the group then to NHI account [KII 004*,* National*,* Lusaka]*

The use of mobile technology would also provide an opportunity to reduce the cost of remitting contributions to the scheme as described by a key informant.

*I think the infrastructure of how to collect money from the point of contributions. People will be making those contributions but how do you move the funds from the contribution’s points to the national health insurance. The ICT infrastructure is better and more convenient than the physical infrastructure because it reduces the cost of remittance. The other way maybe collections through mobile money companies like making bill payments for utilities or school. So people can use the short code on remitting the funds [KII 001*,* National*,* Lusaka]*

Further, most mobile banking technologies were easily accessible and secure. Given that household incomes in rural areas were irregular, leveraging mobile technology would help community members to contribute as and when they had income. A community member from Kalomo narrated.

*It’s a good idea in these villages where we live*,* chances of us having access to money is mostly only once in a year because of the activities which we do of farming. Farming has a time frame where we can farm and sell our produce and get money*,* around December most of us don’t have any money [FGD OO5*,* Kalomo]*

## Community motivation to use saving groups for contribution collection

Motivations relate to the subjective aspects of community members’ behavior, encompassing their interests, attitudes, and perceptions concerning community SGs and the possibility of using them for contribution collection.

### Belonging to savings group yields several social advantages

Community members described several benefits obtained from the SGs. One key takeaway they emphasized was the importance of practicing financial discipline to save. Further, they mentioned that members of the SGs have the option to borrow money from the group, which they can use for sending their children to school and purchasing farming inputs. Some community members explained that the SGs served as transformative agents, leading to improvements in their lives from the time they joined compared to when they were not members. The standard of living in their households, particularly for women, was said to have improved. They gained the ability to make independent financial decisions rather than relying solely on their husbands. A participant of an FGD remarked.

*The benefit I have seen is that I can borrow money from the saving group, they won’t deny me the chance to borrow*,* but if I am not in the saving groups no matter how desperately I need the money*,* no one will lend me the money. But in the saving group I will be able to borrow the money according to the guidelines before the due date of our meetings and able to sort out my problems [FGD 008*,* Kalomo]*

### Community members eagerness to contribute to the scheme to cover illness

The community members expressed their willingness to contribute towards the national health insurance scheme. They mentioned that they would only contribute to the scheme if the premiums were affordable and accommodative. The community members shared that when people fall sick, they often lose their source of income and consequently struggle to afford necessary healthcare services. Therefore, the concept of a prepayment scheme was warmly received. Furthermore, even though some community members were not part of the SGs, they expressed their willingness to make contributions towards the scheme. One FGD participant aptly stated.

*We are agreeing because being sick has no specific time/ period. I can be here meanwhile my child gets sick at home so*,* it will be easy for her to get treatment even if I don’t have money because I would have already insured my family*,* and I think people can join. But if the amount for contributions will not be affordable then we can just opt for cash payment instead of prepayment [FGD 006*,* Choma]*

## Discussion

Our results provide valuable insights into harnessing of community SGs for health insurance contributions in Zambia. The participants underscored the significance of enhanced knowledge about the national health insurance scheme at community level as a key trigger to facilitate engagement of the savings group members. The ability of these groups to be used for contribution collection was strengthened by the presence of a social fund and a collective constitution governing members. However, we identified potential threats, including inconsistent income, mishandling of contributions, and instability within saving groups. Furthermore, the results revealed a promising opportunity to utilize mobile technology to collect contributions from rural communities. Notably, the motivation within the community was underscored by the recognition of social advantages emanating from the savings group and the members’ eagerness to contribute towards healthcare coverage.

We found that the community members were eager to receive more information to enhance their understanding of the national insurance scheme. Our findings align with similar studies that have found a positive association between knowledge and demand for health insurance [15, 32, 33]. In our context, creating awareness about key aspects of the national health insurance scheme among community members during the initial phases of implementation was considered crucial. This was especially important due to the rapid spread of unverified or incorrect information about the scheme among community members. The political nature of health insurance meant people where highly suspicious of the government decision to set it up, making it vulnerable to several interpretations. Incorrect information undermines the trust and confidence that members of the SG may have, potentially making them hesitant to save for their health. Increased awareness about the scheme and its benefits could help community members overcome cultural barriers, foster commitment to the success of the scheme within these SG.

The main abilities in the SGs that could facilitate leveraging them for contribution collection is the availability of a social fund and a collective constitution. The principle of solidarity which underpins insurance stipulates that every insured person receives benefits from the scheme regardless of the premiums paid. The social fund equally functions similarly, providing relief to members of the SGs in times of need. The recognition that there is a social fund that community members can access to mitigate unforeseen emergencies without restrictions encourages them to take this obligation as part of collective responsibility towards their community. Participants from Uganda reported that one of the main reasons that they joined a SG was to increase savings for emergencies, especially health services [34]. In addition, a collective constitution enables leveraging of SGs because it helps ensure that they operate within the confines of the laws, which helps to protect members from any form of exploitation. Further, it contains SGs ‘does’ and ‘don’ts’ as well as responsibility and accountability mechanisms which are vital to ensure appropriate running of the group and effective contributions management. To ensure that these mechanisms are enforced by the constitution, SGs democratically elect their leadership and may sometimes withdraw their support with a vote of no confidence.

Inconsistent incomes, mishandling of contributions and instability are a major threat to the idea of using SGs for contribution collection. Firstly, those with irregular incomes are often at risk of defaulting premium contributions at any given time [35], which may affect the scheme. There could possibly be a disruption of the cohesion within the group owing to the feeling of frustration by some members who see those not contributing, perhaps enjoying similar benefits [36]. Secondly, mishandling of contributions not only results in loss of credibility of the leadership, but also, the group itself to external support stakeholders such as the Ministry of Social services and local NGOs. Ultimately, this may reduce potential support to grow or enhance the health insurance platform within the group. Further, funds mishandling may also imply the inadequacies of the accountability processes. Transparency within these community informal financial groups is crucial if they are to be adopted for health insurance contribution collection. Thirdly, group instability is detrimental because health insurance demands commitment to contribute throughsout a lifetime. Instability may lead to members being apprehensive to commit knowing that the group may not exist for long. Furthermore, instability may overall affect the long-term existence of the group itself, negatively undermining the sustainability of health insurance contribution collection efforts.

An important opportunity that can be harnessed in efforts to adopt savings groups for contribution collection is the use of mobile technologies such as mobile banking facilities. A key characteristic of the SGs is that they are usually made by communities that are in hard-to-reach areas of low socioeconomic activity. Recent government interventions have seen huge growth in mobile and internet penetration rates in Zambia [37, 38]. Our finding implies a major opportunity to overcome geographical barriers and simplify the participation of rural communities in the national health insurance scheme. This is even more critical because complicated illnesses in rural areas are mostly not reported on time till they are advanced is when patients are referred to higher level health facilities for treatment. With the capacity to contribute using mobile platforms, rural households can at least be provided with financial resources using the scheme to cover their treatment costs when referred to higher level facilities for treatment. Additionally mobile technology provides an opportunity streamline health insurance contribution collection with other government systems including pensions and taxes [39].

The social benefits arising from being a member of the SGs facilitates the harnessing of trust, which is a crucial factor for instilling credence to contribute to the health insurance scheme. Furthermore, even when not so many participants were aware of the scheme at the time of data collection, they expressed eagerness to join it when the concept was explained to them. Arising from this, SGs not only provide a platform for engagement but also promoting collective responsivity towards contributions for the insurance scheme [40]. Additionally, they provide a platform to address health related inequalities that are propagated by persistent lack of essential health services amongst poor rural communities [41].

Operationalizing CBOs like SGs as platforms for contribution collection may provide a practical mechanism for expanding coverage among informal sector populations in the NHI scheme in Zambia. SGs could save as a platform for making modest regular payments and these funds could then be periodically remitted to the scheme through a designated group representative. They could also function as community-based enrollment hubs that facilitate collective registration and payment coordination. Linking these arrangements to mobile money systems could enhance transparency and reduce transaction costs and enable real-time verification of payments and membership [42]. However, implementing such models would require regulatory and technological considerations, including establishing accountability mechanisms to safeguard members contributions [35]. Technological barriers such as limited digital infrastructure, challenges in integrating SG records with national health insurance databases and varying digital literacy may affect feasibility.

### Strength and limitations

One of the main strengths of this study is that we were able to collect data from different participant categories. This allowed us to triangulate the different perspectives from the participants giving credence the results we report. Another strength is that we also ensured that the data collection and analysis process was iteratively done between the main author and the research team. For example, the emergent themes relating to the different elements of the study framework were extensively discussed by the research team. A major limitation of this study relates to the fact that we only collected data from rural areas, but we also know that SGs are now common among non-farmer urban informal workers in urban areas, who potentially offer a greater contributive capacity. Although generalizability was not the objective of this qualitative enquiry, we do acknowledge that some of our findings may not exactly apply to other countries with different SG structures. However, we believe that our findings provide credible insights for NHI seeking to establish mechanisms of reaching the informal sector through CBOs.

## Conclusion

Our study emphasizes the pivotal role of enhanced knowledge and understanding of the NHI scheme as a trigger for leveraging community SGs for contribution collection. The eagerness of community members to receive information, highlights the necessity of targeted health insurance awareness campaigns to dispel misinformation and overcome cultural barriers. Furthermore, the presence of a social fund and a collective constitution within SGs are crucial factors for promoting solidarity, collective responsibility, and effective management. Despite threats such as inconsistent member incomes and financial mishandling, opportunities lie in leveraging mobile technology for contribution collection, particularly in rural areas. The community motivation to engage with SGs, driven by social advantages and a collective willingness to contribute, underscores their potential as powerful instruments for facilitating contributions to social health insurance in low-resource communities. Strategic interventions, including financial management measures and the integration of technology, can enhance the potential of leveraging SGs for sustained and inclusive healthcare coverage. Future experimental studies, including randomized control trials, could assess whether the SGs based premium aggregation improves enrollment and payment compliance relative to individual mechanisms, and whether complementary strategies such as digital payments or matched contribution incentives enhance health insurance uptake among informal sector stakeholders.

## Electronic Supplementary Material

Below is the link to the electronic supplementary material.

Supplementary Material 1

## Data Availability

The raw data generated and/or analyzed during the current study are not publicly available but are available from the corresponding author on reasonable request.

## Abbreviations

FGD: Focus group discussion
IDI: In-depth interview
LMICs: Low- middle-income countries
MOTA: Motivation and ability framework
NGOs: Non-governmental organization
NHI: National health insurance
OOP: Out of pocket payment
SG: Savings Group
UHC: Universal health coverage
