# Supplementary material for: Harnessing rural community savings groups for contribution collection in the Zambia national health insurance scheme: an analysis of community perspectives using the motivation and ability framework
Source: BMC Health Serv Res. 2026 May 20;26:981. doi: 10.1186/s12913-026-14721-w (PMC13371017; doi:10.1186/s12913-026-14721-w)
Supplement: Supplementary file 1 — Supplementary Material 1 [file 12913_2026_14721_MOESM1_ESM.docx]

**APPENDIX A: FOCUS GROUP DISCUSSION GUIDE**

**Purpose of the Research:**
The aim of this study is to explore the feasibility of utilizing community informal financial groups for collecting contributions for the national health insurance program. To achieve this, we are interested in hearing your views, suggestions, and recommendations on this topic.

**Discussion of Ground Rules:**
You were selected to participate in this study because you are a member of a savings group. Those who are not members of these groups are not included in the study.

There are no right or wrong answers, only different opinions. We will be recording this discussion, and we ask that only one person speaks at a time.

While it’s not necessary to agree with everyone, please be respectful when others are sharing their views. Regarding cell phones, please put them on silent. If you need to take an urgent call, feel free to step out quietly and return as soon as possible.

My role as the moderator is to guide the discussion, and my assistant will be taking notes. After completing the consent process, we can begin. Please feel comfortable sharing your thoughts freely.

If you encounter any questions, you don’t feel comfortable answering, you are welcome to skip them. Rest assured that all discussions will remain confidential. Do you have any questions before we start the interview?

Turn on the recorder.

I am the interviewer ………………………………

Date ……………………………. Start time …………………… End time ……………….

Background information

Number: Males ………………….… Females……………………

Questions (more questions are likely to arise during the interview)

|  | **MAIN QUESTION** | **PROBE** |
| --- | --- | --- |
| Background information – informal community financial group | | |
|  | What type informal community financial groups are found in this area | - Describe the informal financial groups found in this community? - Of these informal community financial groups which ones are most common? - Explain why you think so–why do people join these informal community financial groups. - What are some of the activities in these informal community financial groups? - What are some of the challenges that people belonging to these informal community financial groups? |
| Awareness of the national health insurance | | |
|  | Kindly explain understanding of the national health insurance scheme (NHI) | - Are you aware of NHI? - If so, what do you understand about its role in provision of health care? - Please explain what possible role the community can play in implementation of the NHI. - The NHI may require that everyone pays for health care in advance by making regular prepayments. - Wound you accept to make mandatory prepayments and explain why you say so |
| Community savings groups | | |
|  |  | - Kindly describe how this community uses the saving groups as informal financial institution. - Describe with examples how you have used the savings group to access healthcare? - Explain how the savings group operate. - Explain how the funds are managed. - Describe your experiences as we members. - What are the benefits of being part of a saving groups? - What are the challenges of being part of the savings group? - Describe the mechanism you have in place to manage the finances in the savings groups. - How do you hold people accountable within the savings groups |
| Feasibility of using community savings groups for contribution collection | | |
|  |  | - What are your views on the possibility of using community savings groups for contribution collection from the community members for the NHI? - How do you think contribution collection can be done through the savings groups? - What is required to be in place? - What are the strengths and weaknesses of saving groups in collecting contribution from the communities? - What are the opportunities and threat to using saving groups in collecting contribution from the communities? - Any recommendations of how feasible it is to use the savings groups for contribution collection |

**APPENDIX B: IN DEPTH INTERVIEWS GUIDE**

**Purpose of the Research:**
The purpose of this research is to assess the feasibility of using community informal financial groups for collecting contributions to the national health insurance program. We are particularly interested in understanding the awareness, demand, implementation, and integration of these programs, as well as gathering your views and recommendations on the topic.

**Discussion of Ground Rules:**
After completing the consent process, we will begin our discussion. You are encouraged to answer all questions, but if any make you uncomfortable, you are free to skip them and move on to the next.

Do you have any questions before we begin the interview?

Turn on the recorders.

I am the interviewer…………………………………, interviewing…………………………… Date ………………………… Start time ………………………. End time ………………… Background information (kindly fill in the information below)

Sex: Male………………………………… Female ………………………….

Tittle ………………………………………………...

Age at last birthday ……………………………………….

Questions (more questions are likely to arise during the interview)

|  | **MAIN QUESTION** | **PROBE** |
| --- | --- | --- |
| Background information – informal community financial groups | | |
|  | What type informal community financial groups are found in this area | - Describe the informal financial groups found in this community? - Of these informal community financial groups which ones are most common? - Explain why you think so–why do people join these informal community financial groups. - What are some of the activities in these informal community financial groups? - What are some of the challenges that people belonging to these informal community financial groups? |
| Awareness of the national health insurance | | |
|  | Kindly explain understanding of the national health insurance scheme (NHI) | - Are you aware of NHI? - If so, what do you understand about its role in provision of health care? - Please explain what possible role the community can play in implementation of the NHI. - The NHI may require that everyone pays for health care in advance by making regular prepayments. - Wound you accept to make mandatory prepayments and explain why you say so |
| Community savings groups | | |
|  |  | - Kindly describe how this community uses the saving groups an informal financial institution. - Describe with examples how you have used the savings group to access healthcare? - Explain how the savings group operate. - Explain how the funds are managed. - Describe your experiences as we members. - What are the benefits of being part of a saving groups? - What are the challenges of being part of the savings group? - Describe the mechanism you have in place to manage the finances in the savings groups. - How do you hold people accountable within the savings groups |
| Feasibility of using community savings groups for contribution collection | | |
|  |  | - What are your views on the possibility of using community savings groups for contribution collection from the community members for the NHI? - How do you think contribution collection can be done through the savings groups? - What is required to be in place? - Would the community be willing to make contribution collection using the savings groups? - What are the strengths and weaknesses of saving groups in collecting contribution from the communities? - What are the opportunities and threat to using saving groups in collecting contribution from the communities? - Any recommendations of how feasible it is to use the savings groups for contribution collection |
